# Morphology and root canal configuration of maxillary lateral incisors: a systematic review and meta-analysis

**DOI:** 10.1038/s41598-024-74026-y

**Published:** 2024-09-28

**Authors:** Thomas Gerhard Wolf, Theodora Rempapi, Richard Johannes Wierichs, Andrea Lisa Waber

**Affiliations:** 1 Department of Restorative, Preventive and Pediatric Dentistry, School of Dental Medicine, University of Bern, Freiburgstrasse 7, CH-3010 Bern, Switzerland; 2 Department of Periodontology and Operative Dentistry, University Medical Center of the Johannes Gutenberg-University Mainz, Mainz, Germany

**Keywords:** Internal morphology, Maxillary lateral incisors, Micro-computed tomography, Physiological foramen geometry, Root canal configuration, Anatomy, Oral anatomy, Dental pulp, Root canal treatment, Dentistry, Endodontics, Pulpitis

## Abstract

The purpose of this study was to explore maxillary lateral incisors (MxLI) intern morphology by analyzing existing literature. We searched five electronical databases (Cochrane, Embase, LILACS, Scopus, MEDLINE via PubMed) using keywords and predefined search terms. Additional studies were identified by cross-referencing and reviewing bibliographies of relevant articles. From 92 initial studies, 27 duplicates were removed, and 65 records screened. After full-text review and hand searching were 19 studies included. The most reported root canal configurations (RCC) of MxLI were Vertucci (Ve) I (1-1-1/1; 78.1–100%), Ve II (2-2-1/1; 0.2–5%), Ve III (1-2-1/1; 0.1–14.6%), Ve IV (2-2-2/2; 0.5%), and Ve V (1-1-2/2; 0.5–4.9%). A meta-analysis of six studies from Europe and Asia indicated sex-differentiated patterns in RCC prevalence: higher occurrences of Ve II (2-2-1/1; OR [95%CI] = 1.19 [0.51, 2.73]), Ve III (1-2-1/1; (OR [95%CI] = 1.72 [0.61, 4.85]), and Ve V (1-1-2/2; (OR [95%CI] = 2.95 [1.02, 8.55]) configurations were noted in males, whereas females predominantly exhibited Ve I (1-1-1/1; [95%CI] = 0.99 [0.97, 1.02]), and Ve IV (2-2-2/2; (OR [95%CI] = 0.11 [0.01, 2.02]). Examination methods varied, with cone beam computed tomography (CBCT) being most commonly (n = 11), followed by staining & clearing (n = 5), and radiographic analysis (n = 1). The predominant RCC in MxLI is type Vertucci I. CBCT is the most common method for assessing the morphology of root canals. However, up to 20% of cases may present with complex and sex-specific patterns, highlighting the need for clinicians to be aware of these differences to prevent complications during endodontic treatments.

## Introduction

The success rate of both nonsurgical orthograde and surgical retrograde endodontic treatment largely depends on a precise three-dimensional comprehension of the internal tooth morphology^1–3^. Variations in morphology can complicate the preparation, disinfection, and obturation of the entire root canal system, which significantly influences treatment outcomes. Thorough knowledge of these morphological features is therefore crucial to the success of endodontic therapy^4,5^. In particular, the diversity of the anatomy of root canals underscores the meaning and need for individualized diagnosis and case evaluation, as a standardized approach does not do justice to complex anatomical conditions. Understanding the prevailing root canal configuration (RCC) is essential to adapting treatment strategies and instruments as well as material selection. Single-rooted maxillary lateral incisors typically present a continuous canal configuration from the pulp chamber root to the apex^1,6–10^. However, studies show that up to twenty percent of these teeth may exhibit anatomical variations that significantly complicate successful treatment and increase the failure risk^7,8^. Such variations often include additional or bifurcating canals, which are not always detected by standard procedures^1,3,8^. This highlights the need for careful preoperative imaging to identify individual treatment challenges. Historically, root canal morphology was studied using *ex vivo* techniques like dye techniques, light microscopy, and conventional radiography, which, while informative, had limitations in visualizing three-dimensional structures^1,6–10^. Recent advancements in imaging, such as cone beam computed tomography (CBCT) and micro-computed tomography (micro-CT), have significantly improved the ability to study root canal morphology, with CBCT used *in vivo* and micro-CT remaining the gold standard for detailed *ex vivo* analysis in research^11,12^. Three-dimensional imaging methods not only provide a more precise representation of the root canal system but also a better recognition of pathological changes that could remain hidden in two-dimensional imaging techniques. Given the complexity and diversity of root canal anatomy/morphology, especially in incisors^13^, it is important to take advantage of both clinical and technological advances to maximize treatment success. A systematic literature review of the anatomical features and RCCs of these teeth can provide precious insights better to understand the challenges and variations in clinical dental practice. The present study therefore aims to analyze the existing literature on the internal morphology and RCCs of maxillary lateral incisors and to shed light on the difficulties that may arise in endodontic treatment. It should help to develop more precise diagnostic and therapeutic approaches that better consider the individual anatomical conditions and thus improve the chances of success of endodontic procedures in the long term.

## Materials and methods

To identify and evaluate the existing studies on intern morphology and root canal configuration (RCC) of maxillary lateral incisors up to March 2024, a systematic review was performed. Databases searched included Embase, Cochrane Library, LILACS, MEDLINE via PubMed, and Scopus, and additional gray literature (www.opengrey.eu). This review follows the PRISMA guidelines^14^, registered in the PROSPERO database (CRD42023394473).

### Selection criteria

In this systematic review the following studies were included: randomized controlled trials, cross-sectional, validation, comparative, and evaluation studies that focused on the internal morphology and RCC of maxillary lateral incisors without restrictions. The search strategy was comprehensive and used both MeSH terms and keywords: (("root canal configuration" OR "root canal system" OR "root canal morphology") AND ((("maxillary lateral incisor" OR "maxillary anterior teeth")) AND (((“morphology” OR “anatomy”))). Additional studies were identified by cross-referencing and manually searching the bibliographies.

Review articles, case reports, and studies that did not specifically address the internal morphology and RCC of maxillary lateral incisors were excluded.

Duplicate studies were removed, and the remaining articles were first screened by two independent reviewers according to title and abstract (T.R., T.G.W.). The relevant articles were subjected to a full-text review by the same reviewers. Details such as authors, year of publication, study origin, sample size, methodology, participant gender, and RCC classifications of Vertucci^1^, Weine et al.^2^, and Briseño Marroquín et al.^3^ were summarized and depicted in a table.

### Quality assessment and risk of bias

The quality assessment of the included RCCs by following the customized quality assessment tool developed by the National Heart, Lung, and Blood Institute (www.nhlbi.nih.gov/health-topics/study-quality-assessment-tools; last access on 17 July 2024) has been assessed by two independent reviewers (A.L.W., T.G.W.). In the event of disagreements between the independent reviewers and if no consensus could be reached, a third reviewer (R.J.W.) was consulted. The risk of bias was assessed by the anatomic quality assessment (AQUA) tool^15^ (T.R., A.L.W.). Disagreements in the assessment of risk of bias were resolved by consulting a third reviewer (T.G.W.).

### Statistical analysis

The meta-analyses of included studies were performed using the Review Manager software (R.J.W.). The statistical analysis for the meta-analyses was performed using Review Manager software (RevMan version 5.4, Cochrane Collaboration, Copenhagen, Denmark, 2014). The odds ratio (OR) was used to determine the effect size. The I^2^ statistic was used to quantify the degree of variability between studies, which was due to heterogeneity rather than chance^16^. Based on the degree of heterogeneity (I^2^ < 35% for low heterogeneity, fixed-effects meta-analysis; I^2^ > 35% for substantial heterogeneity, random-effects meta-analysis), the appropriate meta-analysis model was selected^17,18^. The primary outcome measures comparing various RCCs, sex and geographical factors were presented as odds ratios with 95% confidence intervals (95% CI) for studies with binary outcomes. A *p*-value of 0.05 or less was statistically significant.

## Results

**Fig. 1:**
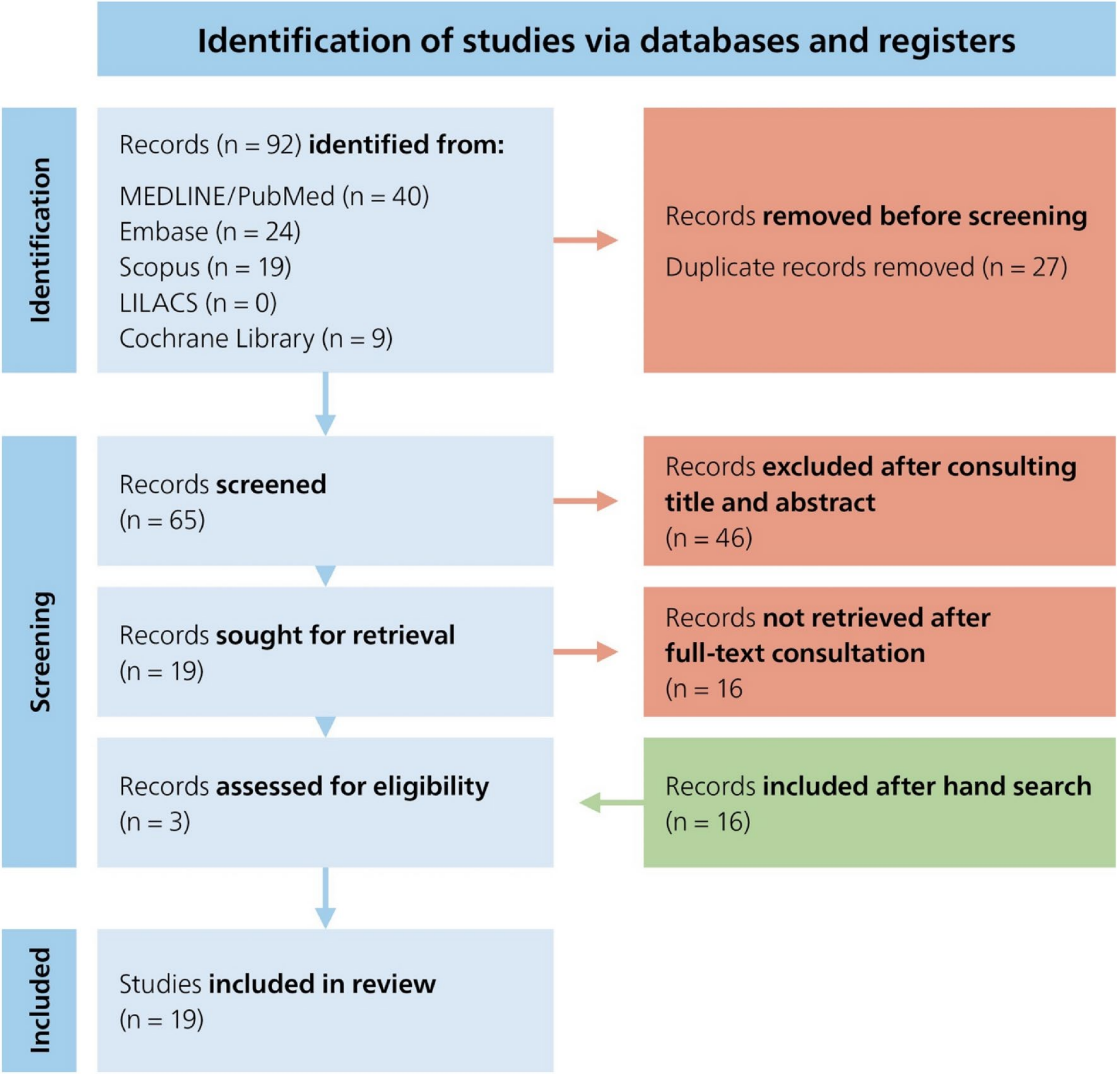
PRISMA flowchart.

**Fig. 2:**
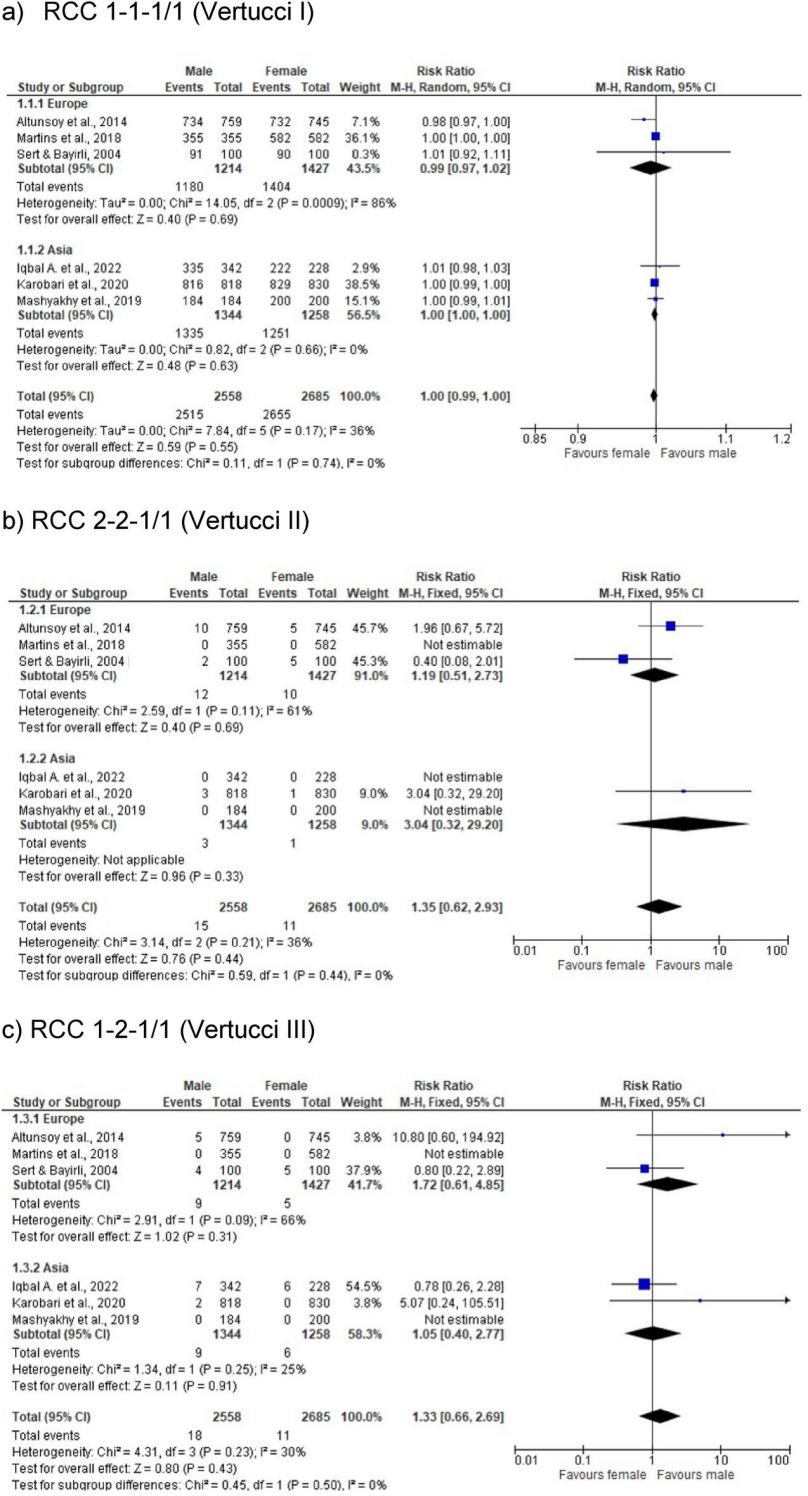
RCC of 1-1-1/1 (Vertucci I), 2-2-1/1 (Vertucci II), and 1-2-1/1 (Vertucci III).

**Fig. 3:**
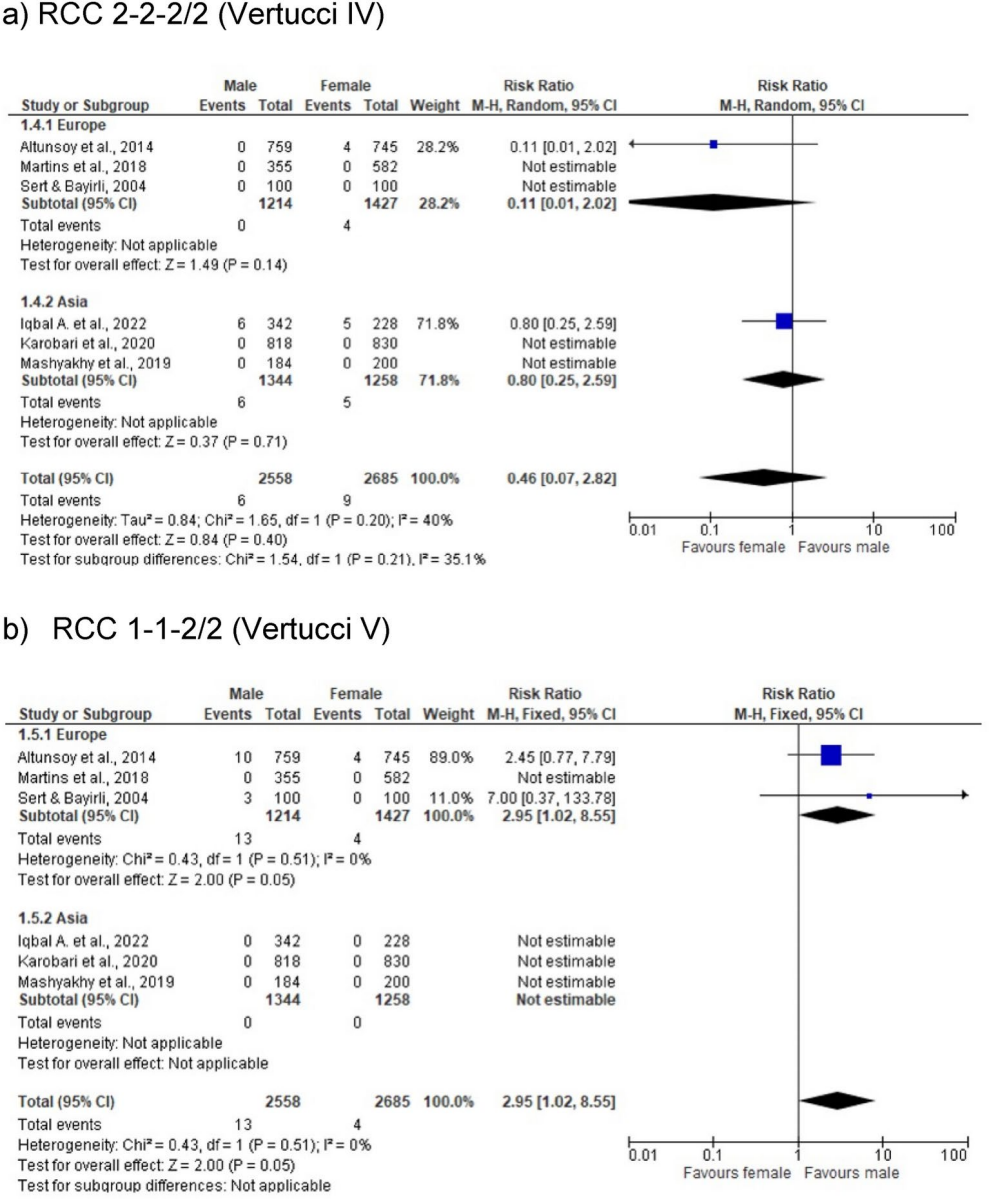
RCC of 2-2-2/2 (Vertucci IV), and 1-1-2/2 (Vertucci V).

The current systematic literature search through five electronic databases resulted in a total of 92 articles. After matching the search results from the different databases, 27 duplicates were removed, and 65 articles screened by the title, and abstract, and later reviewed in full text. Following this process, further articles were considered irrelevant. A hand searching of the bibliographies has identified additional references and finally, 19 articles were included in this review. The selection process is shown in a PRISMA flowchart diagram^14^ (Fig. 1). The meta-analysis results on the sex differences (male, female) according to RCC by Briseño Marroquín et al.^3^ and Vertucci^1^ are shown in Fig. 2 (a. 1-1-1/1 (Vertucci I), b. 2-2-1 /1 (Vertucci II) and c. 1-2-1/1 (Vertucci III) and Fig. 3 (a. 2-2-2/2 (Vertucci IV) and b. 1-1-2/2 (Vertucci V)).

Table 1 shows all references with detailed information on authors, publication year, sample size, sample origin, research method used, and RCC until March 2024. The most commonly RCC is Briseño Marroquín et al.’s 1-1-1/1, also well-known as Weine et al.’s I or Vertucci’s I with a frequency of 78.1–100%^1,6–10,19–31^. The second most common RCC is Briseño Marroquín et al.’s 1-2-1/1 (Vertucci’s III) with 0.1–14.6%^7–10,19,20,28–30^. Further, Briseño Marroquín et al.’s RCC 1-1-2/2 (Vertucci’s V) and Briseño Marroquín et al.’s 2-2-1/1 (Vertucci’s II) occurred with similar frequencies (0.5–4.9% and 0.2–3.5%)^7–10,19,20,29^. Other RCC occurred less frequently as indicated in Table 1. This summary also includes comparative studies where differences between sex^9,23,24^ or different ethnic groups^8,24^ have been investigated. CBCT imaging is the most desstimucribed examination method in the current systematic review (n = 10)^19–29^, followed by the staining and clearing method (n = 5)^1,7–10^. Radiographic analysis was only used in a single report^6^.

**Table 1:** A systematic literature review summary of different morphologic investigations of maxillary lateral incisors.

Report		PP	n	Met	RCC-frequency (%)								
References	Q/R	RCC		Ve (1984)	I	II	III	IV	V	VI	VII	VIII	*
				We (1969)	I	II		III					*
				Br (2015)	1–1-1/1	2-2-1/1	1-2-1/1	2-2-2/2	1-1-2/2	2–1-2/2	1–2-1/2	1–1-3/3	*
Pineda and Kuttler^6^	G	MEX	284	Rx	100.0	–	–	–	–	–	–	–	–
Vertucci^1^	G	USA	100	SC	100.0	–	–	–	–	–	–	–	–
Calişkan et al.^7^	G	TUR	100	SC, MI	78.05	2.44	14.63	–	4.88	–	–	–	–
Sert and Bayirli^9^	G	TUR	100	SC M	91.0	2.0	4.0	–	3.0	–	–	–	–
			100	F	90.0	5.0	5.0	–	–	–	–	–	–
Peiris^8^	G	LKA	88	SC	96.6	–	1.2	–	1.1	–	–	–	1.1
		JAP	66	SC	94.0	–	–	–	4.5	–	–	–	1.5
Weng et al.^10^	G	CHN	70	SC	91.4	2.9	1.4	–	4.3	–	–	–	–
Altunsoy et al.^19^	G	TUR	734	CBCT M	96.7	1.3	0.7	–	1.3	–	–	–	–
			732	CBCT F	98.3	0.7	–	0.5	0.5	–	–	–	–
da Silva et al.^20^	G	BRA	200	CBCT	96.0	3.5	0.5	–	–	–	–	–	–
Monsarrat et al.^21^	G	FRA	191	CBCT	99.0	–	–	–	–	–	–	–	1.0
Martins et al.^22^	G	PRT	902	CBCT	100.0	–	–	–	–	–	–	–	–
Martins et al.^23^	G	PRT	355	CBCT M	100.0	–	–	–	–	–	–	–	–
			582	CBCT F	100.0	–	–	–	–	–	–	–	–
Martins et al.^24^	G	CHN	240	CBCT	100.0	–	–	–	–	–	–	–	–
		WE	937	CBCT	100.0	–	–	–	–	–	–	–	–
Razumova et al.^25^	F	RUS	500	CBCT	100.0	–	–	–	–	–	–	–	–
Pan et al.^26^	G	MYS	362	CBCT	100.0	–	–	–	–	–	–	–	–
Mashyakhy et al.^27^	G	SAU	184	CBCT M	100.0	–	–	–	–	–	–	–	–
			200	CBCT F	100.0	–	–	–	–	–	–	–	–
Nikkerdar et al.^28^	G	IRN	250	CBCT	96.8	–	3.2	–	–	–	–	–	–
Karobari et al.^29^	G	MYS	1651	CBCT	99.7	0.2	0.1	–	–	–	–	–	–
Iqbal et al.^30^	G	SAU	342	CBCT M	98.0	–	2.0	–	–	–	–	–	–
			228	CBCT F	97.4	–	2.6	–	–	–	–	–	–
Buchanan^30^	G	ZAF	403	CBCT	99.5	–	–	–		–	–	–	0.5

CBCT, cone-beam computed tomography; F, fair; G, good; MC, micro-computed tomographic methods; Met, research method used; MI, microscopic; Q/R, quality rank; P, poor; PP, ISO 3166-1 alpha-3 code for countries; R, radiographic; SC, staining and clearing; WE, West European; * no classification given/possible. The summary depicts the sample origin (PP), number and teeth position. The data (mean values) are summarized according to the root canal configurations proposed by Vertucci^1^, Weine et al.^2^, and Briseño Marroquín et al.^3^

## Discussion

The present study aimed to systematically review the literature regarding the maxillary lateral incisors (MxLI) intern morphology and root canal configuration (RCC). An overview for the clinician should be provided to better understand the anatomy of maxillary lateral incisors to be prepared for root canal treatments and lead to superior treatment decisions in endodontic non-surgical as well as surgical treatment.

Briseño Marroquín et al.’s RCC 1-1-1/1 was the most frequent RCC of this systematic review (78.05–100%)^1,6–10,19–31^. The RCC of 1-2-1/1 (0.1–14.63%)^7–10,19,20,28–30^, 1-1-2/2 (0.5–4.88%) and Briseño Marroquín et al.’s RCC 2-2-1/1 (0.2–3.5%) occurred with lower frequencies^7–10,19,20,29^. The circumstance that other RCCs than 1-1-1/1 were less frequently displayed with CBCT scans compared to other methods such as staining and clearing could be explained by the various examination methods. Only one study in this systematic review also examined lateral canals and apical foramina^7^. When lateral canals were observed, they were mostly located in the apical third (12.2%)^7^.

Earlier studies primarily relied on methods like clearing & staining with dye or ink^1,7–10^, magnification^7^, and radiographic imaging^6^, while providing valuable insights, limitations were given in visualizing fine structures like accessory canals and foramina. These methods, though effective for their time, were time-consuming, destructive through e.g. sectioning with a specific slice thickness, and lacked the resolution of modern imaging techniques. Technological advancements such as CBCT and micro-CT have greatly improved the study of root canal morphology^19–31^. While CBCT offers useful *in vivo* imaging, it can be affected by factors like image noise and resolution limitations. In contrast, *ex vivo* methods like staining & clearing (usually together with stereomicroscope approx. 40x) provide detailed views but are destructive and time-consuming^32^. Micro-CT, recognized as the gold standard for *ex vivo* analysis of root canal morphology^12,32^, offers the most reliable, detailed, and noninvasive assessment, preserving tooth structure while using rendering and imaging software to accurately capture and visualize the complexity of internal root canal systems^32–35^. It is possible to follow structures as accessory canals along their entire way from their origination to the end at the accessory foramina. However, to the author’s best knowledge, no micro-CT study has been performed for the internal morphology or RCC of maxillary lateral canals yet.

There are various classification systems to describe the different configurations of RCCs. The most common RCCs have been described by Weine et al.^2^, who differentiated three different RCC types, and Vertucci^1^, who classified eight distinct RCC types. As modern methods like CBCT or micro-CT can imagine increasingly precise structures, the need arose for a classification system that depicted these details. Therefore, the RCC classification according to Briseño Marroquín et al.^3^, which was used in this report, has become established and meets this need for more precise classification, as it defines not only the number of root canals in all thirds of a root but also describes the number of physiological foramina. However, also other precise methods for characterizing the internal tooth morphology have been proposed^36^.

Of the included studies, six compared sex differences^9,19,23,27,29,30^. Besides the previously mentioned research methods used or the ethnic origin, differences in the studies may also be due to sex-specific differences, as these were found when comparing studies from Europe and Asia regarding the frequency of RCC.

Although the age of the investigated individuals was reported in various studies, unfortunately, no analysis was performed. Age is a determining factor for morphological volume changes of the root canal system, but other factors such as wear, caries, occlusal trauma, and the time of eruption also affect the anatomy of the root canal, even if the influence on the morphology of the main root canal may only be minor^8,23,24,37,38^. Nevertheless, it would be interesting to investigate age, which has only been performed in a few such anatomical studies^23,24,38^. Despite the very common RCC of 1-1-1/1 (Ve I) for MxLI, the dental practitioner should always be aware of the complex internal root canal morphology in up to 20% of cases. These may be accessory root canals that cannot be prepared mechanically. This underlines the importance of chemical root canal irrigation. The irrigation protocol and obturation technique should therefore be chosen carefully and errors avoided. Furthermore, the current systematic review provides additional knowledge of RCC and internal morphology of maxillary lateral incisors that could increase the understanding of these structures and thus improve the success of endodontic treatments by clinicians.

## Conclusions

- The most common observed RCC of MxLI are 1-1-1/1 (Vertucci I), 1-2-1/1 (Ve III), 2-2-1/1 (Ve II/We II), and 1-1-2/2 (Ve V)
- The most used method for *in vivo* research on MxLI intern morphology is CBCT
- RCC of 2-2-1/1 (Ve II), 1-2-1/1 (Ve III), and 1-1-2/2 (Ve V) were significantly more often observed in males and 1-1-1/1 (Ve I) and 2-2-2/2 (Ve IV) in females.
- Examination methods varied, with CBCT being the most frequent (n = 11), followed by staining & clearing (n = 5), radiographic analysis (n = 1), and no study using micro-CT

## Data Availability

The authors confirm that the data supporting the findings of this study are available within the article and its supplementary materials.
